# Tardive Peritonitis After Endoscopic Ultrasound-Guided Pancreatic Pseudocyst Drainage: A Case Report

**DOI:** 10.7759/cureus.60179

**Published:** 2024-05-13

**Authors:** Koji Takahashi, Hiroshi Ohyama, Izumi Ohno, Naoya Kato

**Affiliations:** 1 Department of Gastroenterology, Chiba University, Chiba, JPN; 2 Department of Medical Oncology, Chiba University, Chiba, JPN

**Keywords:** stent, endoscopic ultrasound-guided drainage, tardive peritonitis, pancreatic pseudocyst, pancreatic fluid collection

## Abstract

Here, we report a case of tardive peritonitis after endoscopic ultrasound (EUS)-guided transmural pancreatic pseudocyst drainage. A 50-year-old man was diagnosed with acute pancreatitis and a pancreatic pseudocyst measuring 5 cm. Ten months later, his pancreatic pseudocyst was 10 cm. We performed EUS-guided transmural drainage using a lumen-apposing metal stent. After two months, the stent was replaced with a double-pigtail plastic stent. Two months later, the patient developed fever and abdominal pain, and computed tomography revealed abdominal free air. He was diagnosed with peritonitis due to free air caused by a fistula rupture. The double-pigtail plastic stent was removed, and clipping was performed at the fistula site to achieve closure. The patient’s symptoms subsequently improved. Long-term placement of a plastic stent for pancreatic pseudocysts makes recurrence less likely, but late adverse events due to stent placement can occur. Notably, fistula rupture can occur even when the fistula is well-formed several months after the initial drainage.

## Introduction

Pancreatic fluid collections (PFCs), including pancreatic pseudocysts and walled-off pancreatic necrosis, are known complications of acute pancreatitis [1]. Symptomatic PFCs are indicated for treatment with drainage. Drainage approaches to PFCs include endoscopic and percutaneous drainage. In 1992, Grimm et al. reported endoscopic drainage for pancreatic pseudocysts under endoscopic ultrasound (EUS) guidance [2]. Currently, for PFCs that can be accessed through the gastrointestinal tract, EUS-guided transmural drainage is widely performed. Long-term implantation of plastic stents is known to result in low post-treatment relapse rates [3]. However, there is a report of long-term implanted plastic stents causing colorectal perforation [4]. Thus, the long-term course and adverse events after the procedure are often unknown. Here, we describe a case of tardive peritonitis after EUS-guided transmural pancreatic pseudocyst drainage.

## Case presentation

**Figure 1:**
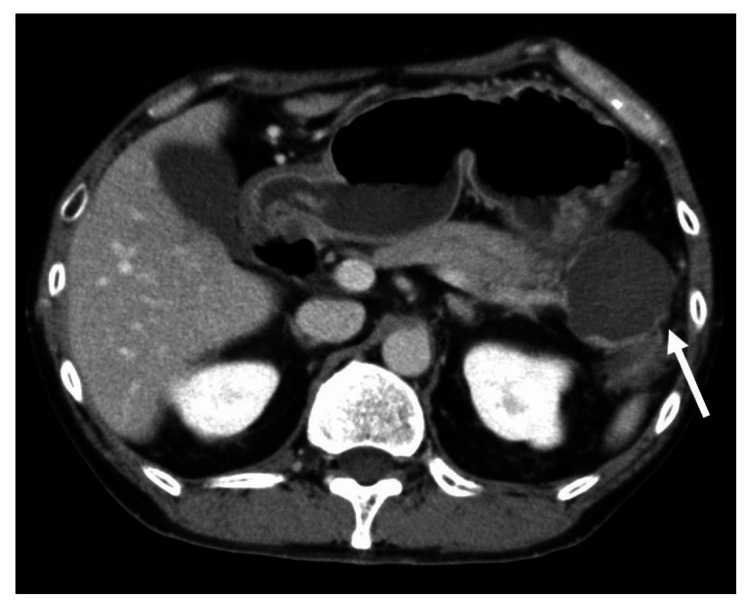
Initial computed tomography. Computed tomography revealed a cyst measuring 5 cm in diameter at the tail of the pancreas (arrow). No bile duct stones were found.

A 50-year-old man had been experiencing persistent abdominal pain for several months and visited a local hospital. He had no notable medical history. Regarding alcohol consumption, he drank approximately 2 liters of beer each day. His blood tests revealed elevated pancreatic enzymes. Contrast-enhanced computed tomography (CT) revealed a cyst measuring 5 cm in diameter at the tail of the pancreas (Figure 1).

**Figure 2:**
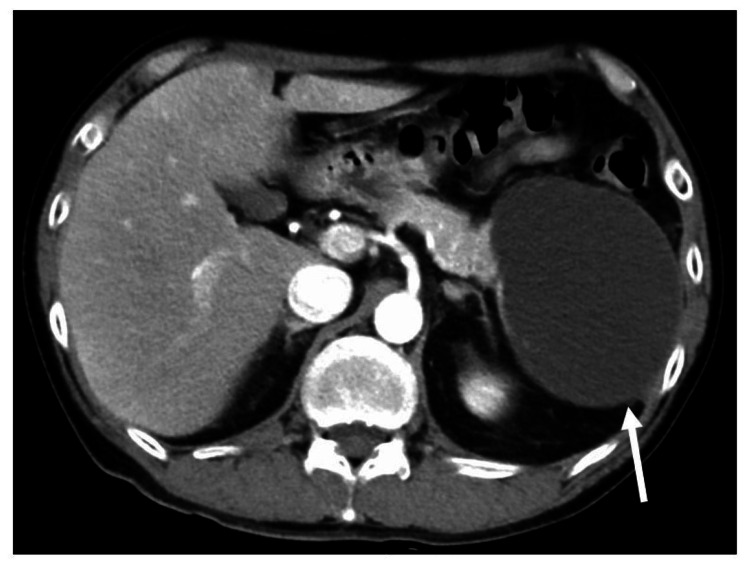
Computed tomography performed 10 months after the initial hospital visit. Computed tomography revealed the pancreatic cyst had grown to 10 cm in diameter (arrow).

No bile duct stones were found. The patient was admitted to the hospital with a diagnosis of acute pancreatitis; his symptoms improved with intravenous treatment, and he was discharged on day 21 after admission. After being discharged from the local hospital, the patient visited our hospital for a detailed examination of the cyst in the pancreatic tail. Contrast-enhanced CT, magnetic resonance cholangiopancreatography (MRCP), and EUS were performed. As a result, we identified the pancreatic cyst as a pseudocyst caused by repeated pancreatitis. He had stopped drinking alcohol since his previous hospital admission. Because he had no symptoms when he visited our hospital, we followed his progress. However, the pancreatic cyst gradually increased in size, and he developed abdominal distension. Ten months after his first visit to our hospital, the pancreatic tail cyst had grown to 10 cm in diameter (Figure 2).

**Figure 3:**
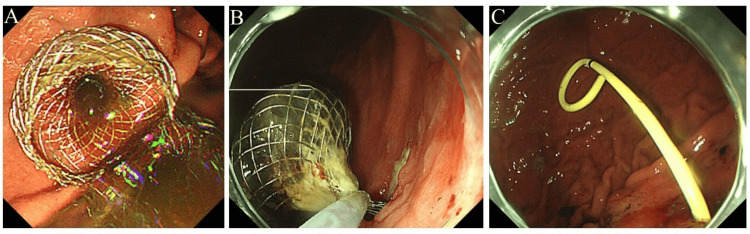
Endoscopic ultrasound-guided transmural pancreatic cyst drainage. We performed endoscopic ultrasound-guided transmural pancreatic cyst drainage. A: The pancreatic cyst was punctured through the stomach under endoscopic ultrasound guidance, and a lumen-apposing metal stent was placed. B: Two months later, the lumen-apposing metal stent was removed. C: A 7 Fr double-pigtail plastic stent was placed.

We decided to perform pancreatic cyst drainage. The pancreatic cyst was punctured through the stomach under EUS guidance, and a lumen-apposing metal stent was placed. Cytology of the aspirated pancreatic cyst fluid revealed no malignant findings. Two months later, the lumen-apposing metal stent was removed, and a 7 Fr double-pigtail plastic stent was placed (Figure 3).

**Figure 4:**
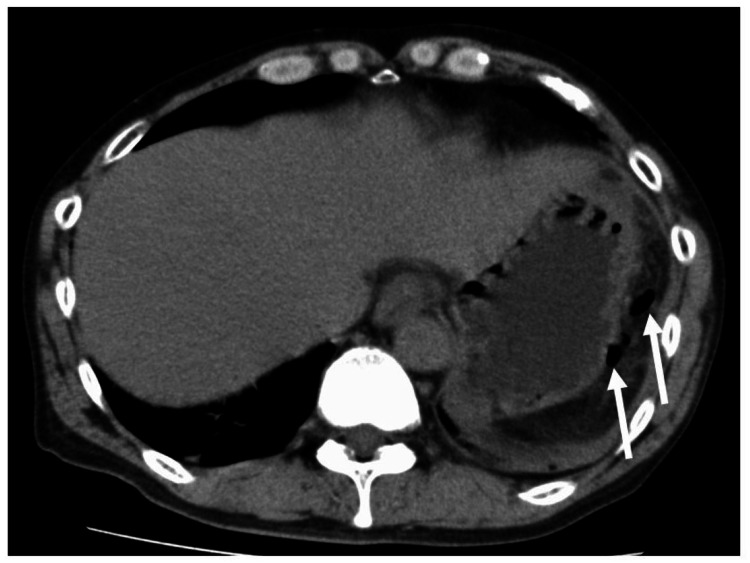
Computed tomography two months after double-pigtail plastic stent placement. Two months after double-pigtail plastic stent placement, fever and abdominal pain developed. Computed tomography revealed abdominal free air (arrow) and increased fat tissue density around the fistula formation site.

After two months, fever and abdominal pain developed. CT revealed free air and increased fat tissue density around the fistula formation site. The patient was diagnosed with peritonitis because of abdominal free air caused by a fistula rupture (Figure 4).

**Figure 5:**
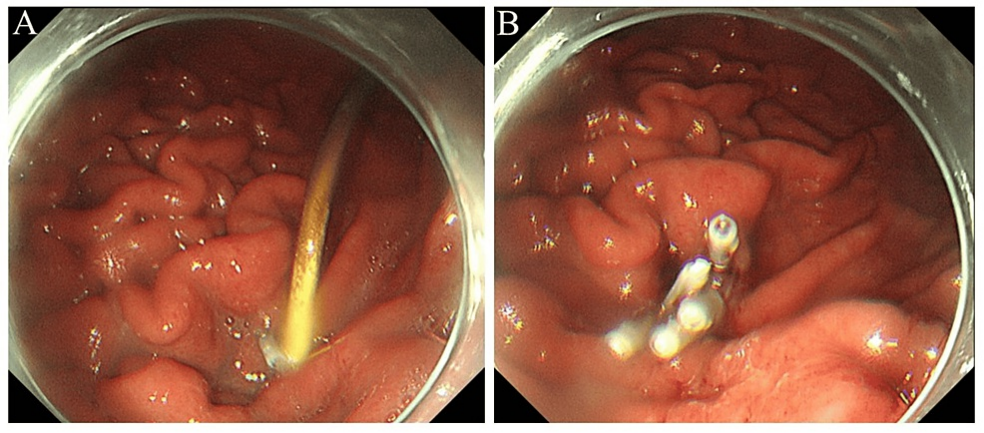
Removal of the double-pigtail plastic stent and clipping. A: A clip was placed near the stent insertion site. B: The double-pigtail plastic stent was removed using an endoscope, and clipping was performed at the fistula site for closure.

The double-pigtail plastic stent was removed using an endoscope, and clipping was performed at the fistula site for closure (Figure 5).

CT on the day after the procedure revealed that the amount of free air in the peritoneal cavity had decreased, and abdominal pain and fever improved. The patient’s progress was uneventful, and he was discharged from the hospital eight days after stent removal. After that, we followed up the patient for one year and found no recurrence of the pancreatic cyst or peritonitis.

## Discussion

Traditionally, plastic stents have been selected as the first stents to be placed for draining PFCs [3,5]. However, recently, metal stents have been widely used [6]. After PFC drainage, long-term stent placement is reported to be effective in preventing recurrences, particularly for patients with disconnected pancreatic duct syndrome [3,7]. Metal stents were reported to be unfit for long-term placement because of the possibility of aneurysm formation or burial [8,9]. Therefore, for patients at a higher risk of recurrence and those for whom long-term stent placement is necessary, metal stents may be removed after short-term placement and replaced with double-pigtail plastic stents [6,10]. However, adverse events owing to long-term plastic stent placement have also been reported. Yamauchi et al. tracked the progress of 36 patients who underwent EUS-guided transmural drainage for symptomatic PFCs. As adverse events related to stent placement, colon perforation due to double-pigtail stent occurred in three cases (8.3%), at 5.8, 17.1, and 33.7 months after double-pigtail stent placement for PFCs [4].

In our case, to prevent the recurrence of PFCs after drainage, a lumen-apposing metal stent was removed after short-term placement and replaced with a double-pigtail stent for long-term placement. Many recurrences of PFC after drainage are related to disconnected pancreatic duct syndrome [5,11,12]. Historically, endoscopic retrograde cholangiopancreatography was considered the gold standard for diagnosing disconnected pancreatic duct syndrome. Currently, secretin-enhanced MRCP is a useful diagnostic method [13]. However, in clinical practice, there are many cases where disconnected pancreatic duct syndrome is not clearly present, but its existence cannot be denied. Secretin-enhanced MRCP was not performed in this case. Based on contrast-enhanced CT, standard MRCP, and EUS, it could not be concluded whether this case had disconnected pancreatic duct syndrome or not.

Few reports of tardive peritonitis after EUS-guided transmural drainage exist. Yasuhara et al. reported on a patient who developed fever and abdominal pain seven days after placing a metal stent using EUS-guided hepaticogastrostomy. Free air was detected on CT, and the patient was diagnosed with tardive peritonitis [14]. In this previously reported case, a fully covered metal stent was placed from the stomach to the intrahepatic bile duct. It is speculated that the gap between the metal stent and the gastric wall or the destruction of the stent cover might have been the cause of peritonitis. In our case, there were no problems with the metal stent for two months, after which the stent was replaced with a plastic stent. In our case, the fistula was considered to be well-formed. It is speculated that tardive peritonitis occurs when the plastic stent moves due to gastric peristalsis or food; therefore, the distal end of the stent injures the fistula or PFC cavity. For drainage of PFCs, careful consideration should be given to whether or not to place a plastic stent when the indwelling metal stent is removed.

## Conclusions

We described a case of tardive peritonitis after EUS-guided pancreatic pseudocyst drainage. Long-term placement of a plastic stent for PFCs makes recurrence less likely, but late adverse events due to stent placement can occur. Notably, fistula rupture can occur even when the fistula is well-formed several months after the initial drainage. The long-term safety of plastic stenting is not known, and there is no consensus on whether the stent should be permanent or removed after a few months to a few years. While there are scattered reports showing the effectiveness of long-term implantation in preventing cyst recurrence, there have also been reports of adverse events. Our case demonstrates that fistula rupture can still occur after fistula formation and serves as an educational case that requires careful follow-up after fistula formation.
